# Supplementary material for: Optimal number of charging station and pricing strategy for the electric vehicle with component commonality considering consumer range anxiety
Source: PLoS One. 2023 May 8;18(5):e0283320. doi: 10.1371/journal.pone.0283320 (PMC10166567; doi:10.1371/journal.pone.0283320)
Supplement: S1 Appendix — (DOCX) [file pone.0283320.s001.docx]

**Appendix**

**Proof of Theorem 1.**

$\frac{\partial^{2}\pi_{1}}{\partial p_{1}^{2}}=-2<0$, $\frac{\partial^{2}\pi_{1}}{\partial p_{2}^{2}}=-2<0$, $\frac{\partial^{2}\pi_{1}}{\partial p_{1}\partial p_{2}}=0$.

Then we get$\left|\begin{matrix} \frac{\partial^{2}\pi_{1}}{\partial p_{1}^{2}} & \frac{\partial^{2}\pi_{1}}{\partial p_{1}\partial p_{2}} \\ \frac{\partial^{2}\pi_{1}}{\partial p_{2}\partial p_{1}} & \frac{\partial^{2}\pi_{1}}{\partial p_{2}^{2}} \end{matrix}\right|=4>0$. Thus, $\pi_{1}$is a joint concave function with $p_{1}$and $p_{2}$. Let $\frac{\partial\pi_{1}}{\partial p_{1}}$, $\frac{\partial\pi_{1}}{\partial p_{2}}=0$, then we can get $p_{1}$and $p_{2}$. Substituting $p_{1}$and $p_{2}$ into$\pi_{1}$and $\pi_{1}$is as follows:

$\pi_{1}=\frac{1}{4}(a_{1}-c^{HH}+\delta r+c\sqrt{e_{1}}+\theta(q_{2}^{H}-1))^{2}+\frac{1}{4}(a_{2}-c^{HL}+\delta r+c\sqrt{e_{1}}+\theta(q_{2}^{L}-1))^{2}-\frac{kr^{2}}{2}-e_{1}$.

$\frac{\partial^{2}\pi_{1}}{\partial r^{2}}=\delta^{2}-k<0$; let $\frac{\partial\pi_{1}}{\partial r}=0$, then we get

$\mathop{r}\limits^{\cdot} =\frac{\delta(a_{1}+a_{2}-c^{HH}-c^{HL}-2\theta+q_{2}^{H}\theta+q_{2}^{L}\theta+2c\sqrt{e_{1}})}{2(k-\delta^{2})}$. Substituting $r$ into $p_{1}$ and $p_{2}$, Theorem 1 is proved.

**Proof of Theorem 2.**

Similar to the proof of Theorem 1, $\frac{\partial^{2}\pi_{2}}{\partial p_{1}^{2}}=-2<0$, $\frac{\partial^{2}\pi_{2}}{\partial p_{2}^{2}}=-2<0$, $\frac{\partial^{2}\pi_{2}}{\partial p_{1}\partial p_{2}}=0$.

Then we get $\left|\begin{matrix} \frac{\partial^{2}\pi_{2}}{\partial p_{1}^{2}} & \frac{\partial^{2}\pi_{2}}{\partial p_{1}\partial p_{2}} \\ \frac{\partial^{2}\pi_{2}}{\partial p_{2}\partial p_{1}} & \frac{\partial^{2}\pi_{2}}{\partial p_{2}^{2}} \end{matrix}\right|=4>0$. Thus, $\pi_{2}$is a joint concave function with $p_{1}$and $p_{2}$. Let $\frac{\partial\pi_{2}}{\partial p_{1}}=0$, $\frac{\partial\pi_{2}}{\partial p_{2}}=0$, then we can get $p_{1}$and $p_{2}$. Substituting $p_{1}$and $p_{2}$ into $\pi_{2}$.

$\frac{\partial^{2}\pi_{2}}{\partial r^{2}}=\delta^{2}-k<0$; let $\frac{\partial\pi_{2}}{\partial r}=0$, then we get

$\mathop{r}\limits^{\cdot\cdot} =\frac{\delta(a_{1}+a_{2}-c^{LH}-c^{LL}-2\theta+q_{2}^{H}\theta+q_{2}^{L}\theta+2c\sqrt{e_{1}})}{2(k-\delta^{2})}$. Substituting $r$ into $p_{1}$ and $p_{2}$, Theorem 2 is proved.

**Proof of Proposition 1.**

$\mathop{r\text{ }}\limits^{\cdot} -\mathop{r\text{ }}\limits^{\cdot\cdot} \text{= -}\frac{2c_{1}\delta(q_{1}^{H2}-q_{1}^{L2})}{2(k-\delta^{2})}<0$；

$\mathop{p_{1}}\limits^{\cdot} -\mathop{p_{1}}\limits^{\cdot\cdot} =\mathop{p_{2}}\limits^{\cdot} -\mathop{p_{2}}\limits^{\cdot\cdot} =\frac{2c_{1}(q_{1}^{H2}-q_{1}^{L2})(k-2\delta^{2})}{4(k-\delta^{2})}$；

When $\sqrt{\frac{k}{2}}<\delta<\sqrt{k}$, we can get $\mathop{p_{1}}\limits^{\cdot} <\mathop{p_{1}}\limits^{\cdot\cdot}$,$\mathop{p_{2}}\limits^{\cdot} \mathop{<p_{2}}\limits^{\cdot\cdot}$; otherwise, if $0<\delta<\sqrt{\frac{k}{2}}$, then $\mathop{p_{1}}\limits^{\cdot} >\mathop{p_{1}}\limits^{\cdot\cdot}$,$\mathop{p_{2}}\limits^{\cdot} \mathop{>p_{2}}\limits^{\cdot\cdot}$;

$\mathop{D_{1}}\limits^{\cdot} -\mathop{D_{1}}\limits^{\cdot\cdot} =\mathop{D_{2}}\limits^{\cdot} -\mathop{D_{2}}\limits^{\cdot\cdot} =-\frac{kc_{1}(q_{1}^{H2}-q_{1}^{L2})}{2(k-\delta^{2})}<0$.

**Proof of Proposition 2.**

$\frac{\partial\mathop{r}\limits^{\cdot} }{\partial\theta}=\frac{\partial\mathop{r}\limits^{\cdot\cdot} }{\partial\theta}=\frac{\delta(q_{2}^{H}+q_{2}^{L}-2)}{2(k-\delta^{2})}<0$;

$\frac{\partial\mathop{p_{1}}\limits^{\cdot} }{\partial\theta}=\frac{\partial\mathop{p_{1}}\limits^{\cdot\cdot} }{\partial\theta}=-\frac{2k-2kq_{2}^{H}+\delta^{2}q_{2}^{H}-\delta^{2}q_{2}^{L}}{4(k-\delta^{2})}<0$;

$\frac{\partial\mathop{p_{2}}\limits^{\cdot} }{\partial\theta}=\frac{\partial\mathop{p_{2}}\limits^{\cdot\cdot} }{\partial\theta}=-\frac{2k(1-q_{2}^{L})-\delta^{2}(q_{2}^{H}-q_{2}^{L})}{4(k-\delta^{2})}<0$;

$\frac{\partial\mathop{D_{1}}\limits^{\cdot} }{\partial\theta}=\frac{\partial\mathop{D_{1}}\limits^{\cdot\cdot} }{\partial\theta}=-\frac{2k(1-q_{2}^{H})+\delta^{2}(q_{2}^{H}-q_{2}^{L})}{4(k-\delta^{2})}<0$;

$\frac{\partial\mathop{D_{2}}\limits^{\cdot} }{\partial\theta}=\frac{\partial\mathop{D_{2}}\limits^{\cdot\cdot} }{\partial\theta}=-\frac{2k(1-q_{2}^{L})-\delta^{2}(q_{2}^{H}-q_{2}^{L})}{4(k-\delta^{2})}<0$.

**Proof of Proposition 3.**

$\frac{\partial\mathop{r}\limits^{\cdot} }{\partial c}=\frac{\partial\mathop{r}\limits^{\cdot\cdot} }{\partial c}=\frac{\delta\sqrt{e_{1}}}{k-\delta^{2}}>0$,

$\frac{\partial\mathop{p_{1}}\limits^{\cdot} }{\partial c}=\frac{\partial\mathop{p_{1}}\limits^{\cdot\cdot} }{\partial c}=\frac{\partial\mathop{p_{2}}\limits^{\cdot} }{\partial c}=\frac{\partial\mathop{p_{2}}\limits^{\cdot\cdot} }{\partial c}=-\frac{\sqrt{e_{1}}(k-2\delta^{2})}{2(k-\delta^{2})}$.

If $0<\delta<\sqrt{\frac{k}{2}}$, then we can get$\frac{\partial\mathop{p_{1}}\limits^{\cdot} }{\partial c}=\frac{\partial\mathop{p_{1}}\limits^{\cdot\cdot} }{\partial c}=\frac{\partial\mathop{p_{2}}\limits^{\cdot} }{\partial c}=\frac{\partial\mathop{p_{2}}\limits^{\cdot\cdot} }{\partial c}>0$. Otherwise, $\mathop{p_{1}}\limits^{\cdot}$,$\mathop{p_{1}}\limits^{\cdot\cdot}$, $\mathop{p_{2}}\limits^{\cdot}$,$\mathop{p_{2}}\limits^{\cdot\cdot}$decrease with $c$.

$\frac{\partial\mathop{D_{1}}\limits^{\cdot} }{\partial c}=\frac{\partial\mathop{D_{2}}\limits^{\cdot} }{\partial c}=\frac{\partial\mathop{D_{1}}\limits^{\cdot\cdot} }{\partial c}=\frac{\partial\mathop{D_{2}}\limits^{\cdot\cdot} }{\partial c}=\frac{k\sqrt{e_{1}}}{2(k-\delta^{2})}>0$.

**Proof of Proposition 4.**

$\frac{\partial\mathop{r}\limits^{\cdot} }{\partial\delta}=\frac{(k\text{+}\delta^{2})(a_{1}+a_{2}-c^{HH}-c^{HL}-2\theta+q_{2}^{H}\theta+q_{2}^{L}\theta+2c\sqrt{e_{1}})}{2(k-\delta^{2})^{2}}>0$;

$\frac{\partial\mathop{p_{1}}\limits^{\cdot} }{\partial\delta}=\frac{\partial\mathop{p_{2}}\limits^{\cdot} }{\partial\delta}=\frac{k\delta(a_{1}+a_{2}-c^{HH}-c^{HL}-2\theta+q_{2}^{H}\theta+q_{2}^{L}\theta+2c\sqrt{e_{1}})}{2(k-\delta^{2})^{2}}>0$;

$\frac{\partial\mathop{r}\limits^{\cdot\cdot} }{\partial\delta}=\frac{(k\text{+}\delta^{2})(a_{1}+a_{2}-c^{LH}-c^{LL}-2\theta+q_{2}^{H}\theta+q_{2}^{L}\theta+2c\sqrt{e_{1}})}{2(k-\delta^{2})^{2}}>0$;

$\frac{\partial\mathop{p_{1}}\limits^{\cdot\cdot} }{\partial\delta}=\frac{\partial\mathop{p_{2}}\limits^{\cdot\cdot} }{\partial\delta}=\frac{k\delta(a_{1}+a_{2}-c^{LH}-c^{LL}-2\theta+q_{2}^{H}\theta+q_{2}^{L}\theta+2c\sqrt{e_{1}})}{2(k-\delta^{2})^{2}}>0$;

$\frac{\partial\mathop{D_{1}}\limits^{\cdot} }{\partial\delta}=\frac{\partial\mathop{D_{2}}\limits^{\cdot} }{\partial\delta}=\frac{k\delta(a_{1}+a_{2}-c^{HH}-c^{HL}-2\theta+q_{2}^{H}\theta+q_{2}^{L}\theta+2c\sqrt{e_{1}})}{2(k-\delta^{2})^{2}}>0$;

$\frac{\partial\mathop{D_{1}}\limits^{\cdot\cdot} }{\partial\delta}=\frac{\partial\mathop{D_{2}}\limits^{\cdot\cdot} }{\partial\delta}=\frac{k\delta(a_{1}+a_{2}-c^{LH}-c^{LL}-2\theta+q_{2}^{H}\theta+q_{2}^{L}\theta+2c\sqrt{e_{1}})}{2(k-\delta^{2})^{2}}>0$.

**Proof of Proposition 5.**

$\frac{\partial\mathop{r}\limits^{\cdot} }{\partial k}=-\frac{\delta(a_{1}+a_{2}-c^{HH}-c^{HL}-2\theta+q_{2}^{H}\theta+q_{2}^{L}\theta+2c\sqrt{e_{1}})}{2(k-\delta^{2})^{2}}<0$;

$\frac{\partial\mathop{p_{1}}\limits^{\cdot} }{\partial k}=\frac{\partial\mathop{p_{2}}\limits^{\cdot} }{\partial k}=-\frac{\delta^{2}(a_{1}+a_{2}-c^{HH}-c^{HL}-2\theta+q_{2}^{H}\theta+q_{2}^{L}\theta+2c\sqrt{e_{1}})}{2(k-\delta^{2})^{2}}<0$;

$\frac{\partial\mathop{r}\limits^{\cdot\cdot} }{\partial k}=-\frac{\delta(a_{1}+a_{2}-c^{LH}-c^{LL}-2\theta+q_{2}^{H}\theta+q_{2}^{L}\theta+2c\sqrt{e_{1}})}{2(k-\delta^{2})^{2}}<0$;

$\frac{\partial\mathop{p_{1}}\limits^{\cdot\cdot} }{\partial k}=\frac{\partial\mathop{p_{2}}\limits^{\cdot\cdot} }{\partial k}=-\frac{\delta^{2}(a_{1}+a_{2}-c^{LH}-c^{LL}-2\theta+q_{2}^{H}\theta+q_{2}^{L}\theta+2c\sqrt{e_{1}})}{2(k-\delta^{2})^{2}}<0$;

$\frac{\partial\mathop{D_{1}}\limits^{\cdot} }{\partial k}=\frac{\partial\mathop{D_{2}}\limits^{\cdot} }{\partial k}=-\frac{\delta^{2}(a_{1}+a_{2}-c^{HH}-c^{HL}-2\theta+q_{2}^{H}\theta+q_{2}^{L}\theta+2c\sqrt{e_{1}})}{4(k-\delta^{2})^{2}}<0$;

$\frac{\partial\mathop{D_{1}}\limits^{\cdot\cdot} }{\partial k}=\frac{\partial\mathop{D_{2}}\limits^{\cdot\cdot} }{\partial k}=-\frac{\delta^{2}(a_{1}+a_{2}-c^{LH}-c^{LL}-2\theta+q_{2}^{H}\theta+q_{2}^{L}\theta+2c\sqrt{e_{1}})}{4(k-\delta^{2})^{2}}<0$.

**Proof of Theorem 3.**

$\frac{\partial^{2}\pi_{3}}{\partial p_{1}^{2}}=-2<0$, $\frac{\partial^{2}\pi_{3}}{\partial p_{2}^{2}}=-2<0$, $\frac{\partial^{2}\pi_{3}}{\partial p_{1}\partial p_{2}}=0$.

Then we get $\left|\begin{matrix} \frac{\partial^{2}\pi_{3}}{\partial p_{1}^{2}} & \frac{\partial^{2}\pi_{3}}{\partial p_{1}\partial p_{2}} \\ \frac{\partial^{2}\pi_{3}}{\partial p_{2}\partial p_{1}} & \frac{\partial^{2}\pi_{3}}{\partial p_{2}^{2}} \end{matrix}\right|=4>0$. Thus, $\pi_{3}$is a joint concave function with $p_{1}$and $p_{2}$. After that, we substitute$p_{1}$and $p_{2}$ into$\pi_{3}$and $\pi_{3}$is as follows:

$\pi_{3}=\frac{1}{4}(a_{1}-c^{HH}+\delta r+c'\sqrt{e_{2}}+\theta(q_{2}^{H}-1))^{2}+\frac{1}{4}(a_{2}-c^{LH}+\delta r+c'\sqrt{e_{2}}+\theta(q_{2}^{H}-1))^{2}-\frac{kr^{2}}{2}-e_{2}$.

$\frac{\partial^{2}\pi_{3}}{\partial r^{2}}=\delta^{2}-k<0$; let $\frac{\partial\pi_{3}}{\partial r}=0$, then we get

$\mathop{r}\limits^{\cdot\cdot\cdot} =\frac{\delta(a_{1}+a_{2}-c^{HH}-c^{LH}-2\theta+2q_{2}^{H}\theta+2c'\sqrt{e_{2}})}{2(k-\delta^{2})}$. Substituting $r$ into $p_{1}$ and $p_{2}$, theorem 3 is proved.

**Proof of Theorem 4.**

Similar to proof of Theorem 3, we could derive Theorem 4.

**Proof of Proposition 6.**

$\mathop{r\text{ }}\limits^{\cdot\cdot\cdot} -\mathop{r\text{ }}\limits^{\cdot\cdot\cdot\cdot} \text{=}-\frac{\delta(q_{2}^{H}-q_{2}^{L})[c_{2}(q_{2}^{H}+q_{2}^{L})-\theta]}{k-\delta^{2}}>0\Rightarrow c_{2}(q_{2}^{H}+q_{2}^{L})-\theta>0$;

$\mathop{r\text{ }}\limits^{\cdot\cdot\cdot} -\mathop{r\text{ }}\limits^{\cdot\cdot\cdot\cdot} \text{=}-\frac{\delta(q_{2}^{H}-q_{2}^{L})[c_{2}(q_{2}^{H}+q_{2}^{L})-\theta]}{k-\delta^{2}}<0\Rightarrow c_{2}(q_{2}^{H}+q_{2}^{L})-\theta<0$;

$\mathop{p_{1}}\limits^{\cdot\cdot\cdot} -\mathop{p_{1}}\limits^{\cdot\cdot\cdot\cdot} =\mathop{p_{2}}\limits^{\cdot\cdot\cdot} -\mathop{p_{2}}\limits^{\cdot\cdot\cdot\cdot} =\frac{(q_{2}^{H}-q_{2}^{L})[c_{2}(k-2\delta^{2})(q_{2}^{H}+q_{2}^{L})+k\theta]}{2(k-\delta^{2})}>0\Rightarrow c_{2}(k-2\delta^{2})(q_{2}^{H}+q_{2}^{L})+k\theta>0$;

$\mathop{p_{1}}\limits^{\cdot\cdot\cdot} -\mathop{p_{1}}\limits^{\cdot\cdot\cdot\cdot} =\mathop{p_{2}}\limits^{\cdot\cdot\cdot} -\mathop{p_{2}}\limits^{\cdot\cdot\cdot\cdot} =\frac{(q_{2}^{H}-q_{2}^{L})[c_{2}(k-2\delta^{2})(q_{2}^{H}+q_{2}^{L})+k\theta]}{2(k-\delta^{2})}<0\Rightarrow c_{2}(k-2\delta^{2})(q_{2}^{H}+q_{2}^{L})+k\theta<0$;

$\mathop{D_{1}}\limits^{\cdot\cdot\cdot} -\mathop{D_{1}}\limits^{\cdot\cdot\cdot\cdot} =\mathop{D_{2}}\limits^{\cdot\cdot\cdot} -\mathop{D_{2}}\limits^{\cdot\cdot\cdot\cdot} =-\frac{k(q_{2}^{H}-q_{2}^{L})[c_{2}(q_{2}^{H}+q_{2}^{L})-\theta]}{2(k-\delta^{2})}>0\Rightarrow c_{2}(q_{2}^{H}+q_{2}^{L})-\theta>0$;

$\mathop{D_{1}}\limits^{\cdot\cdot\cdot} -\mathop{D_{1}}\limits^{\cdot\cdot\cdot\cdot} =\mathop{D_{2}}\limits^{\cdot\cdot\cdot} -\mathop{D_{2}}\limits^{\cdot\cdot\cdot\cdot} =-\frac{k(q_{2}^{H}-q_{2}^{L})[c_{2}(q_{2}^{H}+q_{2}^{L})-\theta]}{2(k-\delta^{2})}<0\Rightarrow c_{2}(q_{2}^{H}+q_{2}^{L})-\theta<0$.

**Proof of Proposition 7.**

$\frac{\partial\mathop{r}\limits^{\cdot\cdot\cdot} }{\partial\theta}=\frac{\delta(q_{2}^{H}-1)}{k-\delta^{2}}<0$;$\frac{\partial\mathop{r}\limits^{\cdot\cdot\cdot\cdot} }{\partial\theta}=\frac{\delta(q_{2}^{L}-1)}{k-\delta^{2}}<0$;

$\frac{\partial\mathop{p_{1}}\limits^{\cdot\cdot\cdot} }{\partial\theta}=\frac{\partial\mathop{p_{2}}\limits^{\cdot\cdot\cdot} }{\partial\theta}\text{=}\frac{k(q_{2}^{H}-1)}{2(k-\delta^{2})}<0$; $\frac{\partial\mathop{p_{1}}\limits^{\cdot\cdot\cdot\cdot} }{\partial\theta}=\frac{\partial\mathop{p_{2}}\limits^{\cdot\cdot\cdot\cdot} }{\partial\theta}\text{=}\frac{k(q_{2}^{L}-1)}{2(k-\delta^{2})}<0$;

$\frac{\partial\mathop{D_{1}}\limits^{\cdot\cdot\cdot} }{\partial\theta}=\frac{\partial\mathop{D_{2}}\limits^{\cdot\cdot\cdot} }{\partial\theta}=\frac{k(q_{2}^{H}-1)}{2(k-\delta^{2})}<0$; $\frac{\partial\mathop{D_{1}}\limits^{\cdot\cdot\cdot\cdot} }{\partial\theta}=\frac{\partial\mathop{D_{2}}\limits^{\cdot\cdot\cdot\cdot} }{\partial\theta}=\frac{k(q_{2}^{L}-1)}{2(k-\delta^{2})}<0$.

**Proof of Proposition 8.**

$\frac{\partial\mathop{r}\limits^{\cdot\cdot\cdot} }{\partial c^{'}}=\frac{\partial\mathop{r}\limits^{\cdot\cdot\cdot\cdot} }{\partial c^{'}}=\frac{\delta\sqrt{e_{2}}}{k-\delta^{2}}>0$;

$\frac{\partial\mathop{p_{1}}\limits^{\cdot\cdot\cdot} }{\partial c^{'}}=\frac{\partial\mathop{p_{2}}\limits^{\cdot\cdot\cdot} }{\partial c^{'}}=\frac{\partial\mathop{p_{1}}\limits^{\cdot\cdot\cdot\cdot} }{\partial c^{'}}=\frac{\partial\mathop{p_{2}}\limits^{\cdot\cdot\cdot\cdot} }{\partial c^{'}}=-\frac{\sqrt{e_{2}}(k-2\delta^{2})}{2(k-\delta^{2})}>0\Rightarrow\frac{k}{\delta^{2}}<2$;

$\frac{\partial\mathop{p_{1}}\limits^{\cdot\cdot\cdot} }{\partial c^{'}}=\frac{\partial\mathop{p_{2}}\limits^{\cdot\cdot\cdot} }{\partial c^{'}}=\frac{\partial\mathop{p_{1}}\limits^{\cdot\cdot\cdot\cdot} }{\partial c^{'}}=\frac{\partial\mathop{p_{2}}\limits^{\cdot\cdot\cdot\cdot} }{\partial c^{'}}=-\frac{\sqrt{e_{2}}(k-2\delta^{2})}{2(k-\delta^{2})}<0\Rightarrow\frac{k}{\delta^{2}}>2$;

$\frac{\partial\mathop{D_{1}}\limits^{\cdot\cdot\cdot} }{\partial c'}=\frac{\partial\mathop{D_{2}}\limits^{\cdot\cdot\cdot} }{\partial c'}=\frac{\partial\mathop{D_{1}}\limits^{\cdot\cdot\cdot\cdot} }{\partial c'}=\frac{\partial\mathop{D_{2}}\limits^{\cdot\cdot\cdot\cdot} }{\partial c'}=\frac{k\sqrt{e_{2}}}{2(k-\delta^{2})}>0$.

**Proof of Proposition 9.**

$\frac{\partial\mathop{r}\limits^{\cdot\cdot\cdot} }{\partial\delta}=\frac{(\delta^{2}+k)(a_{1}+a_{2}-c^{HH}-c^{LH}-2\theta+2q_{2}^{H}\theta+2c'\sqrt{e_{2}})}{2(k-\delta^{2})^{2}}>0$;

$\frac{\partial\mathop{r}\limits^{\cdot\cdot\cdot\cdot} }{\partial\delta}=\frac{(\delta^{2}+k)(a_{1}+a_{2}-c^{HL}-c^{LL}-2\theta+2q_{2}^{L}\theta+2c'\sqrt{e_{2}})}{2(k-\delta^{2})^{2}}>0$;

$\frac{\partial\mathop{p_{1}}\limits^{\cdot\cdot\cdot} }{\partial\delta}=\frac{\partial\mathop{p_{2}}\limits^{\cdot\cdot\cdot} }{\partial\delta}=\frac{\delta k(a_{1}+a_{2}-c^{HH}-c^{LH}-2\theta+2q_{2}^{H}\theta+2c'\sqrt{e_{2}})}{2(k-\delta^{2})^{2}}>0$;

$\frac{\partial\mathop{p_{1}}\limits^{\cdot\cdot\cdot\cdot} }{\partial\delta}=\frac{\partial\mathop{p_{2}}\limits^{\cdot\cdot\cdot\cdot} }{\partial\delta}=\frac{\delta k(a_{1}+a_{2}-c^{HL}-c^{LL}-2\theta+2q_{2}^{L}\theta+2c'\sqrt{e_{2}})}{2(k-\delta^{2})^{2}}>0$;

$\frac{\partial\mathop{D_{1}}\limits^{\cdot\cdot\cdot} }{\partial\delta}=\frac{\partial\mathop{D_{2}}\limits^{\cdot\cdot\cdot} }{\partial\delta}=\frac{\delta k(a_{1}+a_{2}-c^{HH}-c^{LH}-2\theta+2q_{2}^{H}\theta+2c'\sqrt{e_{2}})}{2(k-\delta^{2})^{2}}>0$;

$\frac{\partial\mathop{D_{1}}\limits^{\cdot\cdot\cdot\cdot} }{\partial\delta}=\frac{\partial\mathop{D_{2}}\limits^{\cdot\cdot\cdot\cdot} }{\partial\delta}=\frac{\delta k(a_{1}+a_{2}-c^{HL}-c^{LL}-2\theta+2q_{2}^{L}\theta+2c'\sqrt{e_{2}})}{2(k-\delta^{2})^{2}}>0$.

**Proof of Proposition 10.**

$\frac{\partial\mathop{r}\limits^{\cdot\cdot\cdot} }{\partial k}=-\frac{\delta(a_{1}+a_{2}-c^{HH}-c^{LH}-2\theta+2q_{2}^{H}\theta+2c'\sqrt{e_{2}})}{2(k-\delta^{2})^{2}}<0$;

$\frac{\partial\mathop{r}\limits^{\cdot\cdot\cdot\cdot} }{\partial k}=-\frac{\delta(a_{1}+a_{2}-c^{HL}-c^{LL}-2\theta+2q_{2}^{L}\theta+2c'\sqrt{e_{2}})}{2(k-\delta^{2})^{2}}<0$;

$\frac{\partial\mathop{p_{1}}\limits^{\cdot\cdot\cdot} }{\partial k}=\frac{\partial\mathop{p_{2}}\limits^{\cdot\cdot\cdot} }{\partial k}=-\frac{\delta^{2}(a_{1}+a_{2}-c^{HH}-c^{LH}-2\theta+2q_{2}^{H}\theta+2c'\sqrt{e_{2}})}{4(k-\delta^{2})^{2}}<0$;

$\frac{\partial\mathop{p_{1}}\limits^{\cdot\cdot\cdot\cdot} }{\partial k}=\frac{\partial\mathop{p_{2}}\limits^{\cdot\cdot\cdot\cdot} }{\partial k}=-\frac{\delta^{2}(a_{1}+a_{2}-c^{HL}-c^{LL}-2\theta+2q_{2}^{L}\theta+2c'\sqrt{e_{2}})}{4(k-\delta^{2})^{2}}<0$;

$\frac{\partial\mathop{D_{1}}\limits^{\cdot\cdot\cdot} }{\partial k}=\frac{\partial\mathop{D_{2}}\limits^{\cdot\cdot\cdot} }{\partial k}=-\frac{\delta^{2}(a_{1}+a_{2}-c^{HH}-c^{LH}-2\theta+2q_{2}^{H}\theta+2c'\sqrt{e_{2}})}{4(k-\delta^{2})^{2}}<0$;

$\frac{\partial\mathop{D_{1}}\limits^{\cdot\cdot\cdot\cdot} }{\partial k}=\frac{\partial\mathop{D_{2}}\limits^{\cdot\cdot\cdot\cdot} }{\partial k}=-\frac{\delta^{2}(a_{1}+a_{2}-c^{HL}-c^{LL}-2\theta+2q_{2}^{L}\theta+2c'\sqrt{e_{2}})}{4(k-\delta^{2})^{2}}<0$.
