# Supplementary material for: Optimal number of charging station and pricing strategy for the electric vehicle with component commonality considering consumer range anxiety
Source: PLoS One. 2023 May 8;18(5):e0283320. doi: 10.1371/journal.pone.0283320 (PMC10166567; doi:10.1371/journal.pone.0283320)
Supplement: Attachment — Submitted filename: Response to Reviewers.docx [file pone.0283320.s002.docx]

**Title:** Optimal number of charging station and pricing strategy for the electric vehicle with component commonality considering consumer range anxiety

Reviewer #1:

1. found 2 sub-sections with the same name "Electric vehicle" in the Literature Review, could you please give them a different name or merge the contents into one paragraph.

**Response:** Thank you. We have revised one of the names to "Product commonality".

2. In a table, present the information derived from the formulas.

**Response:** Thank you. According to your suggestion, we have added Table 3 to show the relevant results more clearly.

Reviewer #2:

1. Introduction section needs to be re-written to improve its quality and readability.

**Response:** Thank you. We rewrote the introduction as follows:

‘More and more consumers are beginning to accept electric vehicles, and the sales of electric vehicles have been increasing in recent years. The global electric vehicles sales, including the pure electric vehicles and the plug-in hybrid vehicle, has soared from 2.046 million to 10.65 million from 2019 to 2022 (Chen, 2023 [1]). However, compared with global car sales totaled 81 million in 2022, the EV manufacturer still need make more efforts to let most consumers purchase electric vehicles.

There are two main factors that influence consumer choice: EV price and the range anxiety. The EV price is higher than the gasoline vehicle at least 20% (Wu et al., 2019 [2]). Take Volkswagen's Golf as an example, the pure electric golf is about 22.5 thousand dollars and the gasoline golf with same model is 18.2 thousand dollars. The range anxiety is another obstacle that affects consumer purchasing EVs (Guo et al., 2018 [3], Tanţău & Gavrilescu, 2019 [4], Xu et al., 2020 [5]). Limited battery range and insufficient charging infrastructure can cause inconvenience and range anxiety (Davidov, 2020 [6]).

The EVs’ price is mainly related to the naked vehicle and battery configuration. Using the common parts for multiple models is a good way to reduce the vehicles’ production costs even though the commonality’s R&D still needs invest some money(Heese and Swaminathan, 2006 [7]). For example, in 2021 NIO launched ET5 and ET7 in China，ET5 and ET7 are configured with the same long life battery (the longest range is 1,000 kilometers per charge) and different naked vehicle. Tesla produced Model Y that has two configurations-- the standard battery life version and the long range version with the same naked vehicle. Note that the NIO and Tesla chose different components for the two products as the commonality--NIO chose the battery, Tesla chose the naked vehicle. Motivated by the two manufacturers’ commonality configuration, we try to explore how should EV manufacturers choose common parts, and the impact of the selection of common parts on product prices, sales and corporate profits.

Range anxiety is mainly divided into limited battery range and inconvenient charging station. At present, many EV manufacturers have increased their investment in battery research and development to increase battery range (Ke and Chen, 2022 [8]，Chen and Fan, 2020 [9]). For example, BYD invested 1.5 billion dollars to build a 40GWh new battery production line. In addition, some manufacturers, such as Tesla, have begun to establish their own brand charging stations to improve the charging convenience, in order to increase EVS’ sales. Both investing in battery R&D and constructing the charging stations will increase the cost and price of electric vehicles. By balancing the costs of battery R&D and the charging station’s construction and the revenue from increased sales, how the EV manufacturer should configure the battery and whether and how many charging stations should be constructed are the significant problems worth studying.

Based on above EV manufacturer’s commonality selection, the battery R&D and charging station construction reality, we mainly focus on following questions:

(1) Which components (battery or naked vehicle) should be used as a commonality when the EV manufacturers provides different types of EVs? And how does the manufacturer set the commonality quality level, i.e., high or low quality?

(2) Whether the manufacturer should construct charging station? If yes, how many charging stations should be constructed?

(3) How the component commonality’s quality and the cost coefficient of the charging stations affect EV prices?

In order to address above questions, we assume that the EV manufacturer provides two types of EVs and discuss two basic scenarios: using battery and naked vehicle as common parts for the two types of EVs, respectively. And in each scenario, we consider the common part’s quality could be high or low. For each scenario, we present the optimal number of the charging stations and EVs’ prices, then we compare the optimal solutions and discuss effects of commonality on manufacturer’s decisions in different scenarios. Further, we present the EV manufacturer’s optimal commonality strategy by numerical simulations. Finally, we give the managerial insights.

Our paper mainly has three contributions. First, we divide the range anxiety into battery range anxiety and charging convenience anxiety and both of them affect the EV demand, while most other studies only consider the one type of anxiety (Davidov, 2020 [6], Guo et al., 2018 [3], Luo et al., 2020 [10], Tanţău and Gavrilescu, 2019 [4], Xu et al., 2020 [5], Zhang et al., 2021 [11]). Analyzing the range anxiety in detail will allow manufacturers to take more targeted measures to boost EV demand and make more profits. Second, we incorporate the component commonality into EV manufacturer’s decision process, while most other studies focus on how to choose battery quality or how to set the number of the charging stations (Bi et al., 2019 [12], Bonges and Lusk, 2016 [13], Csiszár, 2019 [14], Zhu et al., 2018 [15]). The commonality design is an important decision for automobiles, especially considering the high cost of developing a production line (about 20 million per product line for vehicles). Third, we discuss the mutual effect between improving battery range and increasing the number of the charging stations. Understanding the relationship between the two measures will avoid the EV manufacturer spend extra money on easing range anxiety and make more profit.

The structure of this paper is as follows: In Section 2, we give the literature review. In Section 3, we present the model formulation. In Section 4, we present the four scenarios with different commonalities and provide the optimal solutions. Then we compare the different scenarios and give the suggestions for the manufacturer. In Section 5, we conclude our research and give the future research orientations.’

2. What is the motivation of the proposed work? Research gaps, objectives of the proposed work should be clearly justified

**Response:** Thank you. We present the motivation in Section 1:

‘More and more consumers are beginning to accept electric vehicles, and the sales of electric vehicles have been increasing in recent years. The global electric vehicles sales, including the pure electric vehicles and the plug-in hybrid vehicle, has soared from 2.046 million to 10.65 million from 2019 to 2022 (Chen, 2023 [1]). However, compared with global car sales totaled 81 million in 2022, the EV manufacturer still need make more efforts to let most consumers purchase electric vehicles.

There are two main factors that influence consumer choice: EV price and the range anxiety. The EV price is higher than the gasoline vehicle at least 20% (Wu et al., 2019 [2]). Take Volkswagen's Golf as an example, the pure electric golf is about 22.5 thousand dollars and the gasoline golf with same model is 18.2 thousand dollars. The range anxiety is another obstacle that affects consumer purchasing EVs (Guo et al., 2018 [3], Tanţău & Gavrilescu, 2019 [4], Xu et al., 2020 [5]). Limited battery range and insufficient charging infrastructure can cause inconvenience and range anxiety (Davidov, 2020 [6]).

The EVs’ price is mainly related to the naked vehicle and battery configuration. Using the common parts for multiple models is a good way to reduce the vehicles’ production costs even though the commonality’s R&D still needs invest some money(Heese and Swaminathan, 2006 [7]). For example, in 2021 NIO launched ET5 and ET7 in China，ET5 and ET7 are configured with the same long life battery (the longest range is 1,000 kilometers per charge) and different naked vehicle. Tesla produced Model Y that has two configurations-- the standard battery life version and the long range version with the same naked vehicle. Note that the NIO and Tesla chose different components for the two products as the commonality--NIO chose the battery, Tesla chose the naked vehicle. Motivated by the two manufacturers’ commonality configuration, we try to explore how should EV manufacturers choose common parts, and the impact of the selection of common parts on product prices, sales and corporate profits.

Range anxiety is mainly divided into limited battery range and inconvenient charging station. At present, many EV manufacturers have increased their investment in battery research and development to increase battery range (Ke and Chen, 2022 [8]，Chen and Fan, 2020 [9]). For example, BYD invested 1.5 billion dollars to build a 40GWh new battery production line. In addition, some manufacturers, such as Tesla, have begun to establish their own brand charging stations to improve the charging convenience, in order to increase EVS’ sales. Both investing in battery R&D and constructing the charging stations will increase the cost and price of electric vehicles. By balancing the costs of battery R&D and the charging station’s construction and the revenue from increased sales, how the EV manufacturer should configure the battery and whether and how many charging stations should be constructed are the significant problems worth studying.’

Our research gaps are also presented in Section 1:

‘First, we divide the range anxiety into battery range anxiety and charging convenience anxiety and both of them affect the EV demand, while most other studies only consider the one type of anxiety (Davidov, 2020 [6], Guo et al., 2018 [3], Luo et al., 2020 [10], Tanţău and Gavrilescu, 2019 [4], Xu et al., 2020 [5], Zhang et al., 2021 [11]). Analyzing the range anxiety in detail will allow manufacturers to take more targeted measures to boost EV demand and make more profits. Second, we incorporate the component commonality into EV manufacturer’s decision process, while most other studies focus on how to choose battery quality or how to set the number of the charging stations (Bi et al., 2019 [12], Bonges and Lusk, 2016 [13], Csiszár, 2019 [14], Zhu et al., 2018 [15]). The commonality design is an important decision for automobiles, especially considering the high cost of developing a production line (about 20 million per product line for vehicles). Third, we discuss the mutual effect between improving battery range and increasing the number of the charging stations. Understanding the relationship between the two measures will avoid the EV manufacturer spend extra money on easing range anxiety and make more profit.’

Objectives of the proposed work are to determine how the manufacturer selects common components and determines the quality level of common components. Besides，to find out if and how many charging stations the manufacturer needs to build. Finally, to determine the impact of the quality of common components and the cost coefficient of charging station construction on the price of electric vehicles.

3. Overall, the basic background is not introduced well, where the notations are not illustrated much clear.

**Response:** Thank you. We have rewritten the introduction and given the background in the introduction:

‘More and more consumers are beginning to accept electric vehicles, and the sales of electric vehicles have been increasing in recent years. The global electric vehicles sales, including the pure electric vehicles and the plug-in hybrid vehicle, has soared from 2.046 million to 10.65 million from 2019 to 2022 (Chen, 2023 [1]). However, compared with global car sales totaled 81 million in 2022, the EV manufacturer still need make more efforts to let most consumers purchase electric vehicles.

There are two main factors that influence consumer choice: EV price and the range anxiety. The EV price is higher than the gasoline vehicle at least 20% (Wu et al., 2019 [2]). Take Volkswagen's Golf as an example, the pure electric golf is about 22.5 thousand dollars and the gasoline golf with same model is 18.2 thousand dollars. The range anxiety is another obstacle that affects consumer purchasing EVs (Guo et al., 2018 [3], Tanţău & Gavrilescu, 2019 [4], Xu et al., 2020 [5]). Limited battery range and insufficient charging infrastructure can cause inconvenience and range anxiety (Davidov, 2020 [6]).

The EVs’ price is mainly related to the naked vehicle and battery configuration. Using the common parts for multiple models is a good way to reduce the vehicles’ production costs even though the commonality’s R&D still needs invest some money(Heese and Swaminathan, 2006 [7]). For example, in 2021 NIO launched ET5 and ET7 in China，ET5 and ET7 are configured with the same long life battery (the longest range is 1,000 kilometers per charge) and different naked vehicle. Tesla produced Model Y that has two configurations-- the standard battery life version and the long range version with the same naked vehicle. Note that the NIO and Tesla chose different components for the two products as the commonality--NIO chose the battery, Tesla chose the naked vehicle. Motivated by the two manufacturers’ commonality configuration, we try to explore how should EV manufacturers choose common parts, and the impact of the selection of common parts on product prices, sales and corporate profits.

Range anxiety is mainly divided into limited battery range and inconvenient charging station. At present, many EV manufacturers have increased their investment in battery research and development to increase battery range (Ke and Chen, 2022 [8]，Chen and Fan, 2020 [9]). For example, BYD invested 1.5 billion dollars to build a 40GWh new battery production line. In addition, some manufacturers, such as Tesla, have begun to establish their own brand charging stations to improve the charging convenience, in order to increase EVS’ sales. Both investing in battery R&D and constructing the charging stations will increase the cost and price of electric vehicles. By balancing the costs of battery R&D and the charging station’s construction and the revenue from increased sales, how the EV manufacturer should configure the battery and whether and how many charging stations should be constructed are the significant problems worth studying.’

4. The literature has to be strongly updated with some relevant and recent papers focused on the fields dealt with in the manuscript.

**Response:** Thank you. We have added the following latest literature to the literature review section.

Guo et al. (2023) analyzed that compared with fuel vehicles, electric vehicles have no cost advantage due to the high purchase cost. Ke & Chen (2022) find consumer trust positively affects the demand of the electric vehicles using the manufacturer-made batteries and then affects the manufacturer's battery R&D decision. Kondev et al. (2023) showed that when the vehicle is more powerful, electric vehicles have a price advantage over internal combustion engine vehicles.

Guo X, Sun Y, Ren D. Life cycle carbon emission and cost-effectiveness analysis of electric vehicles in China[J]. Energy for Sustainable Development, 2023, 72: 1-10.

Ke H, Chen X. Battery R&D decision of electric vehicle manufacturer considering government subsidy[J]. Kybernetes, 2022 (ahead-of-print).

Kondev B, Dixon J, Zhou Z, et al. Putting the foot down: Accelerating EV uptake in Kyrgyzstan[J]. Transport Policy, 2023, 131: 87-96.

5. The study lacks a theoretical framework which is important for the reader to grasp the crust of the research.

**Response:** Thank you. We added Figure 1 to better present the research content of this paper.

Fig. 1. Supply chain structure under different scenarios

6. Explain why the current method was selected for the study, its importance and compare with traditional methods.

**Response:** Thank you. In this paper, we used optimization theory to solve the model which is a general method in decision-making progress. We did not improve the method. The main contributions of this article are the following three aspects.

‘First, we divide the range anxiety into battery range anxiety and charging convenience anxiety and both of them affect the EV demand, while most other studies only consider the one type of anxiety (Davidov, 2020 [6], Guo et al., 2018 [3], Luo et al., 2020 [10], Tanţău and Gavrilescu, 2019 [4], Xu et al., 2020 [5], Zhang et al., 2021 [11]). Analyzing the range anxiety in detail will allow manufacturers to take more targeted measures to boost EV demand and make more profits. Second, we incorporate the component commonality into EV manufacturer’s decision process, while most other studies focus on how to choose battery quality or how to set the number of the charging stations (Bi et al., 2019 [12], Bonges and Lusk, 2016 [13], Csiszár, 2019 [14], Zhu et al., 2018 [15]). The commonality design is an important decision for automobiles, especially considering the high cost of developing a production line (about 20 million per product line for vehicles). Third, we discuss the mutual effect between improving battery range and increasing the number of the charging stations. Understanding the relationship between the two measures will avoid the EV manufacturer spend extra money on easing range anxiety and make more profit.’

Also, the linear demand functions are adopted in this paper. Because the linear structure well delineate the impact of price, battery quality and number of charging stations on demand. Besides, linear models are widely used in marketing and supply chain management literature due to their parsimony and tractability for analysis and the results derived from linear models can also be applied for other demand forms that can be approximated or locally approximated by linear functions.

7. Authors are suggested to include more discussion on the results and also include some explanation regarding the justification to support why the proposed method is better in comparison towards other methods

**Response:** Thank you. According to your suggestion, we have revised the discussion as follows:

In this subsection, we will compare the four scenarios and discuss in which scenario the manufacturer could make more money. Considering the complexity of the profit functions, we use the numerical examples to give the comparison.

The different common components for the manufacturer implies different unit product cost reduction and production line R&D investment. Hence, we discuss the effects of the cost saving of different common components on the optimal selection of the manufacturer's production line. Let $a_{1}=a_{2}=2000$,$e_{1}=800$,$e_{2}=1000$,$c_{1}=1000$,$c_{2}=2000$,$k=20000$,$\delta=20$. In Fig. 6, $q_{1}^{H}=0.8$,$q_{1}^{L}=0.5$,$q_{2}^{L}=0.5$,$\theta=1200$. In Figs. 8 and 9, $q_{1}^{H}=0.5$,$q_{1}^{L}=0.2$,$q_{2}^{H}=0.6$,$q_{2}^{L}=0.3$.

Fig. 6. Partion of the $c-c'$ as $q_{2}^{H}=0.7$.

Fig. 6 shows that when the cost reduction coefficient of the battery as the common component is relatively big, using battery as the common component can make the manufacturer get more profit (Zone I and II). When the cost reduction coefficient of using battery as the common component is very small, using naked vehicle as the common component may make the manufacturer get more profit (Zone III, IV and V). And the cost saving coefficient of battery common parts has greater influence on the selection of commonality. In general, low quality commonality could make manufacturers gain more profits. When the cost reduction coefficient of using battery as the common component and using naked vehicle as the common component are at two extremes, the manufacturer then should choose suitable components as common components rather than considering the quality selection of components.

Fig. 7. Partion of the $c-c'$ as $\theta=1200$. Fig. 8. Partion of the $c-c'$ as $\theta=2200$.

Figs. 7 and 8 present the impact of consumer range anxiety towards battery on manufacturers' optimal profit. When consumers' range anxiety level is very low, choosing low-quality naked vehicles or battery as common components could get more profit. However, when consumers’ range anxiety towards battery is very high, manufacturers should choose low-quality naked vehicles or high-quality battery as common components to maximize profits. This is just in line with the reality that the sales volume of Wuling Hongguang mini EVs with low naked vehicle quality and Tesla with high battery quality rank among the top three in the world. In sum, it is more likely to obtain high profits by using low-quality naked vehicle as commonality.

8. Does this kind of study have never attempted before? Justify this statement and give an appropriate explanation to do so in this paper.

**Response:** Thank you. Some scholars have done similar research before, but our research takes a new perspective ‘First, we divide the range anxiety into battery range anxiety and charging convenience anxiety and both of them affect the EV demand, while most other studies only consider the one type of anxiety (Davidov, 2020 [6], Guo et al., 2018 [3], Luo et al., 2020 [10], Tanţău and Gavrilescu, 2019 [4], Xu et al., 2020 [5], Zhang et al., 2021 [11]). Analyzing the range anxiety in detail will allow manufacturers to take more targeted measures to boost EV demand and make more profits. Second, we incorporate the component commonality into EV manufacturer’s decision process, while most other studies focus on how to choose battery quality or how to set the number of the charging stations (Bi et al., 2019 [12], Bonges and Lusk, 2016 [13], Csiszár, 2019 [14], Zhu et al., 2018 [15]). The commonality design is an important decision for automobiles, especially considering the high cost of developing a production line (about 20 million per product line for vehicles). Third, we discuss the mutual effect between improving battery range and increasing the number of the charging stations. Understanding the relationship between the two measures will avoid the EV manufacturer spend extra money on easing range anxiety and make more profit.’

9. Improve the conclusion section

**Response:** Thank you. We have revised the conclusion as follows

‘5 Conclusion

Assume one manufacturer provides two types of EVs, in this paper we divide electric vehicles into two parts--naked vehicle and battery, to explore the component commonality choice, and the naked vehicle and battery quality configuration. In addition, considering consumers’ sensitivity towards the charging convenience, we also discuss the optimal number of the charging station built by the EV manufacturer.

5.1 Managerial insights

We obtain some interesting managerial insights: first, high consumers’ range anxiety towards battery will induce high demands and prices of EV with high quality battery. Hence, facing high consumer range anxiety, manufacturers should produce EV with high-quality battery. As the anxiety decreases, then the demands of the EV with high quality battery lower. When the anxiety towards battery is very low, then the prices and demands of the product with low quality battery are even bigger than those with high quality battery as the number of the charging stations increases. Therefore, when the EV manufacturer determines the EVs’ battery configuration, identifying consumer range anxiety towards battery in the target market is very significant.

Second, the larger consumers’ sensitivity towards charging station and range anxiety towards battery, the higher EV configuration and prices. When consumers are very concerned about the battery range and charging convenience, the manufacturer needs provide the high-end electric vehicles using the high quality naked vehicle as commonality, such as Tesla. If consumers don't have much anxiety about mileage, for example, they purchase EV as a short distance courtesy car, then the manufacturer could configure the EVs using low quality naked vehicle as the common part, such as Hongguang mini EV. Hence, before the EV manufacturer enters to the market, it is very important to determine the product position and configuration.

Third, the low quality naked vehicle as common component will increase both the number of the charging stations and the demand. This is a good way to increase the number of charging stations, that is, increasing the sales of low-end electric vehicles to drive the construction of charging stations. Hence, the government could encourage the manufacturer to develop low-end EVs that have huge potential market demand to stimulate the charging station’s construction.

Fourth, if one manufacturer who produces EVs with low quality naked vehicle and builds the charging station may ask for much higher EV price than the manufacturer who provides the high-end EV and does not construct charging station, which depends on the demand and cost increment incurred by increasing one charging station. For example, when the increased demand is relatively large and the cost is not very big incurred by one more charging station, then the low-end EVs producing by one manufacturer will have a higher price than the high-end EVs providing by the other manufacturer.

Finally, using the component commonality will increase both EVs demands and the number of the charging stations. But the EVs prices may not decrease as the number of the charging stations increases, which depends on the relationship between the demand increment incurred by one more charging station and the cost coefficient of building the charging station. In addition, there is no absolute profit advantage in making battery as common parts or naked vehicle as common parts. The choice of the common part is determined by the production cost coefficient saved by the common part, the R&D cost of the common part, the production cost of batteries and naked vehicle, and the consumers' mileage anxiety and so on. When consumers’ range anxiety towards battery is very high, manufacturers should choose low-quality naked vehicles or high-quality battery as common components. The low quality naked vehicle as common component will increase both the number of the charging stations and the demand and the manufacturer is more likely to obtain high profits.’

10. What are the limitations of the study?

**Response:** Thank you. The limitations of the study are as follows: In this paper, we only discuss the number of the charging stations built by the manufacturer, but do not consider the charging station built by the government or the third party, hence future study could investigate how the public charging infrastructure affects manufacturer’s decisions. In addition, the number of the charging station and the EV demand interact with each other, but in this paper, we only incorporate the effects of the number of charging station on demand and do not consider how the demand increment affects the number of the number of the charging station. Above two limitations point out the two future research directions.
